# Skirted Prosthetic Valves Are Associated With Increased Risk of Subclinical Leaflet Thrombosis Following Transcatheter Aortic Valve Replacement: A Multicenter Study

**DOI:** 10.31083/RCM49694

**Published:** 2026-09-11

**Authors:** Yifan Yang, Qiang Li, Jing Yao, Yunfeng Yan, Moyang Wang, Xinmin Liu, Fei Yuan, Zhengming Jiang, Taiyang Luo, Qian Zhang, Yongjian Wu, Guangyuan Song

**Affiliations:** ^1^Interventional Center of Valvular Heart Disease, Beijing Anzhen Hospital, Capital Medical University, 100029 Beijing, China; ^2^Department of Cardiology, Fuwai Hospital, National Center for Cardiovascular Disease, Chinese Academy of Medical Science and Peking Union Medical College, 100037 Beijing, China

**Keywords:** subclinical leaflet thrombosis, hypo-attenuated leaflet thickening, transcatheter aortic valve replacement, self-expanding valves

## Abstract

**Background:**

Subclinical leaflet thrombosis (SLT) is associated with an increased risk of thromboembolic events, valve dysfunction, and mortality in patients undergoing transcatheter aortic valve replacement (TAVR). However, data on the incidence and risk factors for SLT in patients undergoing TAVR using self-expanding valves (SEVs) remain limited.

**Methods:**

This study included patients who underwent TAVR using SEVs and received a postoperative four-dimensional computed tomography scan within six months at Beijing Anzhen Hospital and Fuwai Hospital. SLT was defined as the presence of hypo-attenuated leaflet thickening (HALT) with or without reduced leaflet motion (RLM), and characterized by increased leaflet thickness affecting one or more leaflets. Univariate and multivariable logistic regression analyses were conducted to explore the relationship between variables and the occurrence of SLT.

**Results:**

A total of 311 patients (mean age 72.02 ± 8.23 years, 52.4% males) were included in the analysis. HALT was detected in 91 patients (29.3%), while RLM was observed in 45 patients (14.5%). Most HALT (81.3%) and RLM (73.3%) cases were classified as grades 1–2. Multivariable logistic regression analysis revealed that using skirted prosthetic valves was significantly associated with SLT (adjusted odds ratio: 2.999; 95% confidence interval: 1.250–7.397; *p* = 0.015).

**Conclusions:**

Over one-quarter of patients undergoing TAVR using SEVs developed SLT within six months. The use of skirted prosthetic valves was independently associated with SLT.

## 1. Introduction

Transcatheter aortic valve replacement (TAVR) has emerged as a safe and effective treatment for severe aortic stenosis. Current guidelines on valvular heart disease recommend TAVR as a reasonable option for patients aged >70 years and for those at high surgical risk [1].

Subclinical leaflet thrombosis (SLT) can be observed on cardiac computed tomography (CT) in 10%–30% of patients undergoing TAVR [2,3,4]. Isolated leaflet thrombosis is known as hypo-attenuated leaflet thickening (HALT) on four-dimensional computed tomography (4D-CT). If it decreases leaflet motion, it is referred to as reduced leaflet motion (RLM). Recent studies have raised concerns about SLT, suggesting that it may pose a risk of thromboembolic events, valve dysfunction, and mortality [5,6,7]. As the indications for TAVR expand to younger and lower surgical-risk patients, it has become increasingly critical to ensure the long-term durability and functionality of implanted TAVR prostheses. Although the mechanism of SLT is not fully understood, studies suggest that blood turbulence and stasis at the sinus of Valsalva and around the prosthetic valve are potential contributing factors [8,9,10].

In China, self-expanding valves (SEVs) are the most commonly used type of valve in TAVR. SEVs vary in features such as valve size, tapering, and the presence of a skirt. These features might influence blood flow dynamics, potentially leading to SLT [11,12]. However, data on the incidence and risk factors for SLT in patients undergoing TAVR using SEVs remain limited.

Therefore, the aim of this study was to investigate the incidence and potential risk factors associated with SLT among patients undergoing TAVR using SEVs.

## 2. Materials and Methods

### 2.1 Study Population

This multicenter retrospective study was conducted on patients with severe aortic stenosis who underwent TAVR at Beijing Anzhen Hospital and Fuwai Hospital, Beijing, CN, from May 1, 2021 to March 24, 2023. Written informed consent was obtained from each subject for the anonymous use of their data. A total of 311 patients who underwent TAVR using SEVs and received a postoperative 4D-CT scan within six months were included in the final analysis after excluding patients with the following conditions: (a) valve-in-valve; (b) balloon-expandable valve; (c) insufficient CT quality for analysis (Fig. 1).

**Fig. 1.**
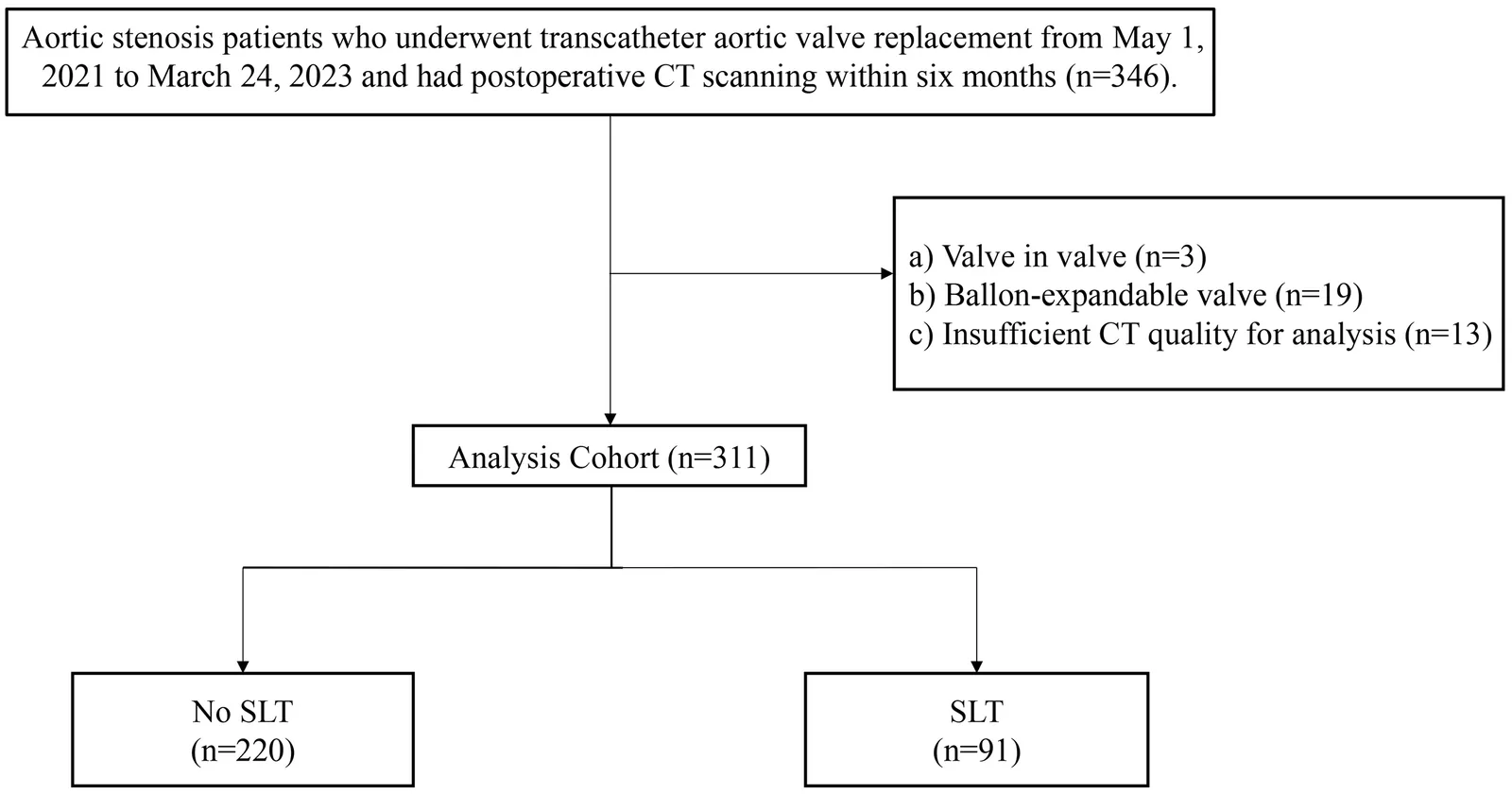
**Study flowchart**. Abbreviations: CT, computed tomography; SLT, subclinical leaflet thrombosis.

TAVR procedures were performed by an experienced structural heart team, which included structural heart cardiologists, an echocardiologist, and an anesthesiologist. Before TAVR, all patients underwent a comprehensive pre-procedural evaluation, including structural and peripheral CT angiography, echocardiography, and coronary angiography.

### 2.2 Data Collection

Clinical data for all enrolled patients were retrieved from the Beijing Anzhen Hospital and Fuwai Hospital databases. These data comprised demographic characteristics, laboratory test results, details of the TAVR procedure, echocardiography, and CT information. Following the TAVR procedure, most patients underwent a pre-discharge echocardiogram and review by the structural heart team. Subsequent follow-ups were typically conducted during outpatient visits at one and six months post-TAVR. These visits included a comprehensive clinical review, encompassing a thorough history and physical examination. Additionally, patients underwent echocardiography to evaluate the prosthetic valve function and a CT scan to check for the presence of SLT and to assess frame morphology.

### 2.3 CT Evaluation and Image Analysis

CT images were reconstructed and analyzed using the 3mensio Structural Heart software (version 10.3, build 5493.20; Pie Medical Imaging B.V., Maastricht, the Netherlands) by two experienced CT readers. The prosthetic valve leaflets were meticulously evaluated throughout the diastolic period. SLT was defined as the presence of HALT with or without RLM, characterized by increased leaflet thickness (hypo-attenuated opacity attached to the valve) affecting one or more leaflets, assessed through two-dimensional multiplanar reconstructions [13,14]. RLM was defined as the presence of reduced leaflet mobility or motion in 4D-CT. In discordant interpretations, a third experienced CT reader was consulted for adjudication. For 4D-CT images in which SLT is detected, its location, length and thickness were further assessed and described. HALT was graded according to its extent along the arcuate leaflet, and RLM was graded according to the degree of restriction in leaflet motion [14].

The valve types used in the present study and their classification according to the presence or absence of skirt are summarized in **Supplementary Table 1**. The structural distinction between valves with and without an outer sealing skirt is schematically shown in Fig. 2. Prosthesis eccentricity was calculated using the formula: [(Dmax – Dmin) / Dmax × 100%] [15]. The nominal diameter and area of the prosthetic valve were provided by the valve company according to the size used in the patients. The taper of the transcatheter heart valve was defined as [2 × Aortic valve waist area / (Aortic valve inflow tract area + Aortic valve outflow tract area) × 100%] [16].

**Fig. 2.**
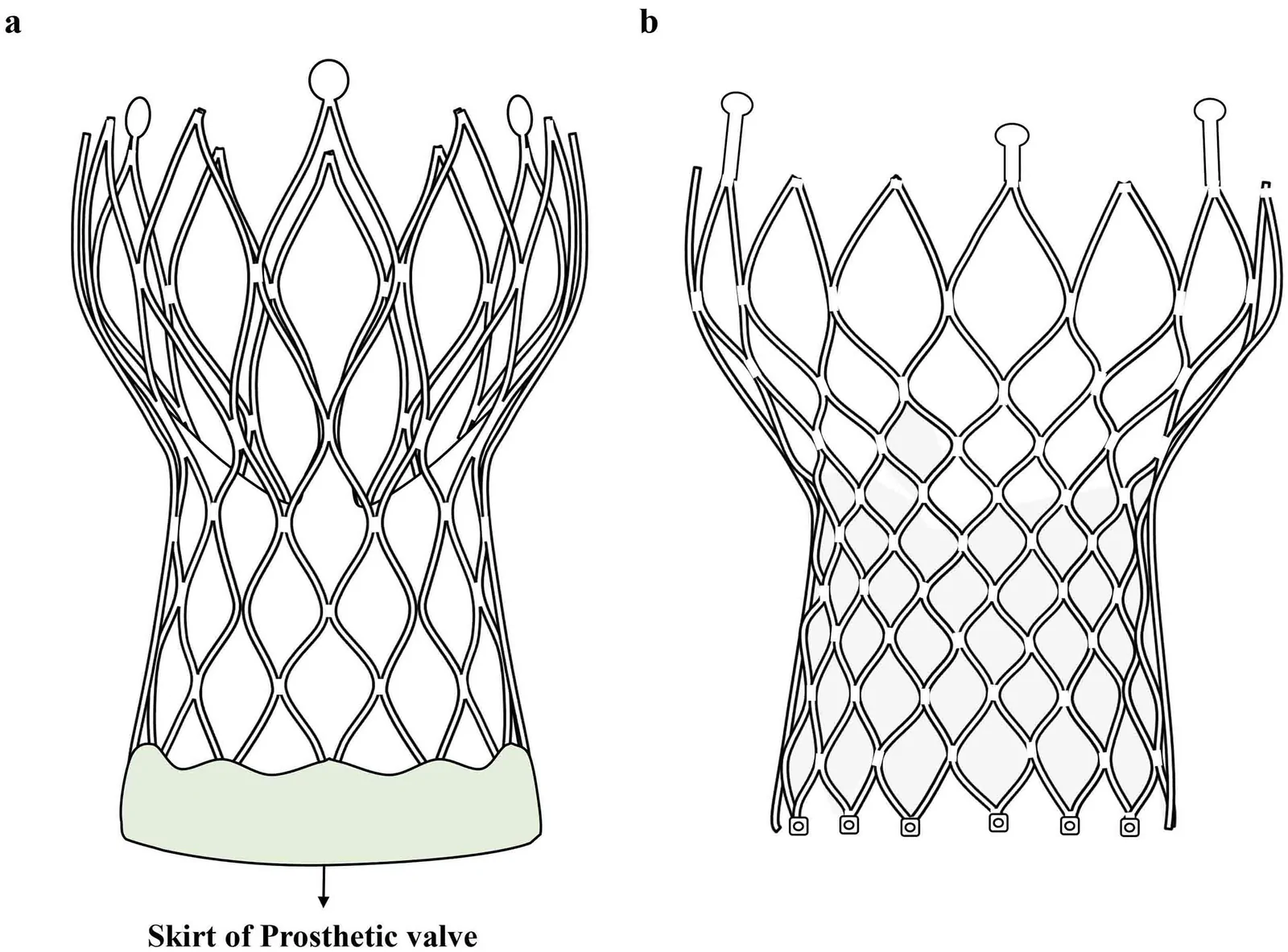
**Comparison between prosthetic valves with and without skirt**. (a) Prosthetic valves with skirt. (b) Prosthetic valves without skirt.

### 2.4 Statistical Analysis

The study population was divided into SLT and no SLT groups. Continuous variables are presented as the mean with standard deviation, or the median with interquartile range. Categorical variables are presented as counts and percentages (%). Pearson’s chi-squared test was used to compare categorical variables, and the *t*-test was employed for continuous variables. Univariable and multivariable logistic regression analyses were conducted to assess the association between potential risk factors and SLT. Variables that showed a statistically significant association in univariate analysis and have significant clinical implications were subsequently included in a multivariate logistic regression model to identify independent factors of SLT. Multicollinearity among the variables included in the multivariable model was assessed using the variance inflation factor. To investigate whether the relationship between skirted prosthetic valves and SLT is influenced by paravalvular leakage (PVL), Doppler echocardiography and color-flow imaging were used to perform a semi-quantitative grading of PVL. The definition and measurement criteria for PVL are based on the three-tier classification system outlined in Valve Academic Research Consortium 3 and the American Society of Echocardiography guidelines [17,18]: (1) mild (<10%), (2) moderate (10%–30%), and (3) severe (>30%). Mild paravalvular leakage is considered positive for paravalvular leakage, the population was categorized into four groups: prosthetic valve without a skirt and not combined with PVL, prosthetic valve without a skirt and combined with PVL, prosthetic valve with a skirt and not combined with PVL, and prosthetic valve with a skirt and combined with PVL. Statistical analyses were conducted using R software (version 4.3.3; R Foundation for Statistical Computing, Vienna, Austria). For all analyses, a two-sided *p*-value < 0.05 indicated statistical significance.

## 3. Results

### 3.1 Clinical Characteristics

A total of 311 patients (mean age 72.02 ± 8.23 years, 52.4% males) were included in the final analysis. Among these, 175 (56.3%) had coronary artery disease, 85 (27.3%) had diabetes mellitus, and 179 (57.6%) had hypertension (Table 1). Patients were categorized based on post-TAVR CT imaging into the SLT group (n = 91, 29.3%) and the no SLT group (n = 220, 70.7%). RLM was present in 45 patients (14.5%) (Fig. 3).

**Table 1.** **Baseline clinical characteristics of the patients**.

				Total cohort (N = 311)	No SLT (N = 220)	SLT (N = 91)	*p*-value
Demographics							
	Age (years)			72.02 (8.23)	71.64 (8.82)	72.95 (6.57)	0.201
	STS score			7.71 (2.68)	7.63 (2.73)	8.14 (2.46)	0.582
	Female, n (%)			148 (47.59)	109 (49.55)	39 (42.86)	0.342
	Body mass index (kg/m^2^)			24.67 (4.05)	24.69 (3.80)	24.62 (4.61)	0.905
	Diabetes mellitus, n (%)			85 (27.33)	56 (25.45)	29 (31.87)	0.284
	Hypertension, n (%)			179 (57.56)	128 (58.18)	51 (56.04)	0.906
	Hyperlipidemia, n (%)			127 (40.84)	91 (41.36)	36 (39.56)	0.925
	Atrial fibrillation, n (%)			57 (18.33)	43 (19.55)	14 (15.38)	0.508
	Coronary artery disease, n (%)			175 (56.27)	127 (57.73)	48 (52.75)	0.561
	Percutaneous coronary intervention, n (%)			39 (12.54)	23 (10.45)	16 (17.58)	0.063
	Coronary artery bypass grafting, n (%)			3 (0.96)	3 (1.36)	0 (0.00)	0.674
	NYHA functional class						0.722
		II		28 (9.00)	18 (8.18)	10 (10.99)	
		III		184 (59.16)	132 (60.00)	52 (57.14)	
		IV		99 (31.83)	70 (31.82)	29 (31.87)	
Biochemical test							
	Alb (g/L)			41.11 (4.49)	41.14 (4.39)	41.01 (4.77)	0.838
	Cr (µmol/L)			85.89 (27.95)	84.89 (29.02)	88.34 (25.17)	0.380
	LDL (mmol/L)			2.35 (0.86)	2.39 (0.86)	2.26 (0.86)	0.295
	NT-pro-BNP (pg/mL), median [IQR]			1231.00 [390.75, 3903.50]	1240.00 [404.50, 4083.50]	1177.00 [372.50, 3630.50]	0.501
TTE							
	LVEF (%)			57.23 (11.81)	56.99 (12.03)	57.82 (11.32)	0.570
	LVEDD (mm)			51.87 (8.83)	51.75 (9.02)	52.16 (8.41)	0.710
	LVESD (mm)			35.98 (9.24)	35.75 (9.30)	36.58 (9.12)	0.502
	Mean aortic valve gradient (mmHg)			50.24 (24.24)	51.38 (24.42)	47.29 (23.66)	0.216
	Peak aortic valve gradient (mmHg)			84.28 (39.75)	85.97 (40.49)	79.84 (37.64)	0.256
	Moderate or severe MR, n (%)			112 (36.01)	78 (35.45)	34 (37.36)	0.850
	Moderate or severe TR, n (%)			55 (17.68)	39 (17.73)	16 (17.58)	1.000
CT variables before TAVR							
	Bicuspid aortic valve, n (%)			150 (48.23)	111 (50.45)	39 (42.86)	0.273
	Aortic valve annulus area (mm^2^)			484.95 (109.42)	481.00 (111.41)	495.28 (104.06)	0.337
	Aortic valve calcium score			322.00 [60.00, 581.00]	326.00 [70.00, 637.50]	318.00 [57.75, 514.75]	0.429
Medication at discharge							
	Antiplatelet therapy, n (%)			217 (69.77)	156 (70.91)	61 (67.03)	0.682
		Antiplatelet therapy type					
			None, n (%)	94 (30.22)	64 (29.09)	30 (32.97)	
			Single antiplatelet therapy, n (%)	109 (35.05)	77 (35.00)	32 (35.16)	
			Dual antiplatelet therapy, n (%)	108 (34.73)	79 (35.91)	29 (31.87)	
	Anticoagulation therapy, n (%)			78 (25.08)	62 (28.18)	16 (17.58)	0.076
		Anticoagulation therapy type					
			None, n (%)	233 (74.92)	158 (71.82)	75 (82.42)	
			Direct oral anticoagulant, n (%)	72 (23.15)	58 (26.36)	14 (15.38)	
			Warfarin, n (%)	6 (1.93)	4 (1.82)	2 (2.20)	

Abbreviations: Alb, albumin; Cr, creatinine; CT, computed tomography; SLT, subclinical leaflet thrombosis; LDL, low-density lipoprotein; MR, mitral regurgitation; NT-pro-BNP, N-terminal pro–B-type natriuretic peptide; TAVR, transcatheter aortic valve replacement; TR, tricuspid regurgitation; STS, Society of Thoracic Surgeons Score; NYHA, New York Heart Association; IQR, interquartile range; LVEF, left ventricular ejection fraction; LVEDD, left ventricular end-diastolic dimension; LVESD, left ventricular end-systolic diameter; TTE, transthoracic echocardiography.

**Fig. 3.**
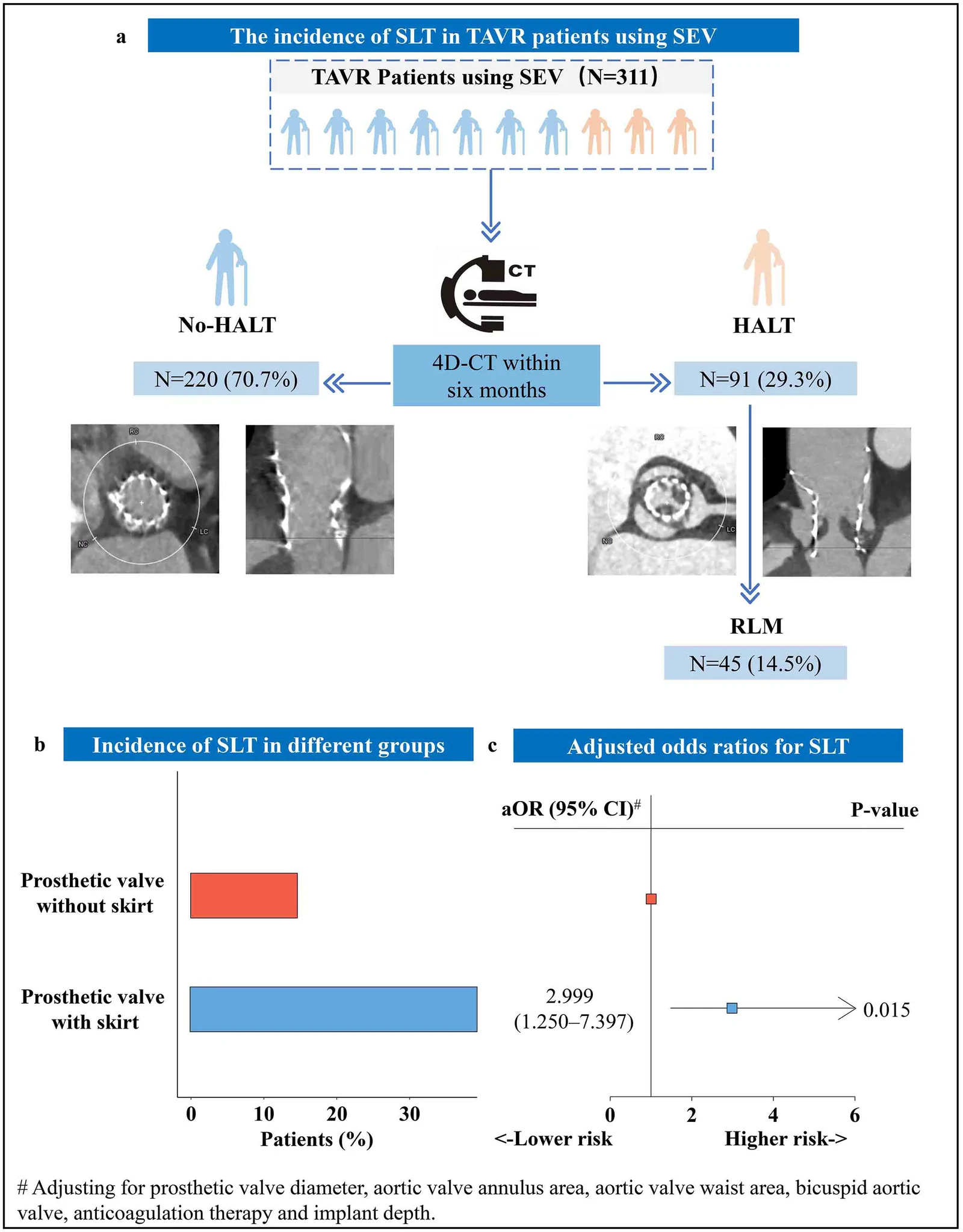
**Incidence and risk factors of SLT in transcatheter aortic valve replacement patients using self-expanding valves**. (a) The incidence of SLT in TAVR patients using SEVs. (b) The incidence of SLT in prosthetic valve without skirt group and prosthetic valve with skirt group. (c) Adjusted odds ratios of prosthetic valve with skirt for SLT. Abbreviations: aOR, adjusted odds ratio; CI, confidence interval; HALT, hypo-attenuated leaflet thickening; RLM, reduced leaflet motion; SEV, self-expanding valves; SLT, subclinical leaflet thrombosis; TAVR, transcatheter aortic valve replacement.

Among the 91 patients with HALT, 52 (57.1%) were classified as grade 1, 22 (24.2%) as grade 2, 11 (12.1%) as grade 3, and 6 (6.6%) as grade 4. Among the 45 patients with RLM, 17 (37.8%) were classified as grade 1, 16 (35.6%) as grade 2, 10 (22.2%) as grade 3, and 2 (4.4%) as grade 4. With regard to the distribution of leaflet thrombosis, 53 thrombotic leaflets (38.7%) had leaflet thrombosis at the non-coronary cusp, 52 (38.0%) at the right coronary cusp, and 32 (23.4%) at the left coronary cusp. The number of leaflet thrombosis present in one leaflet, two leaflets, and three leaflets was 50 (54.9%), 36 (39.6%), and 5 (5.5%), respectively (Fig. 4).

**Fig. 4.**
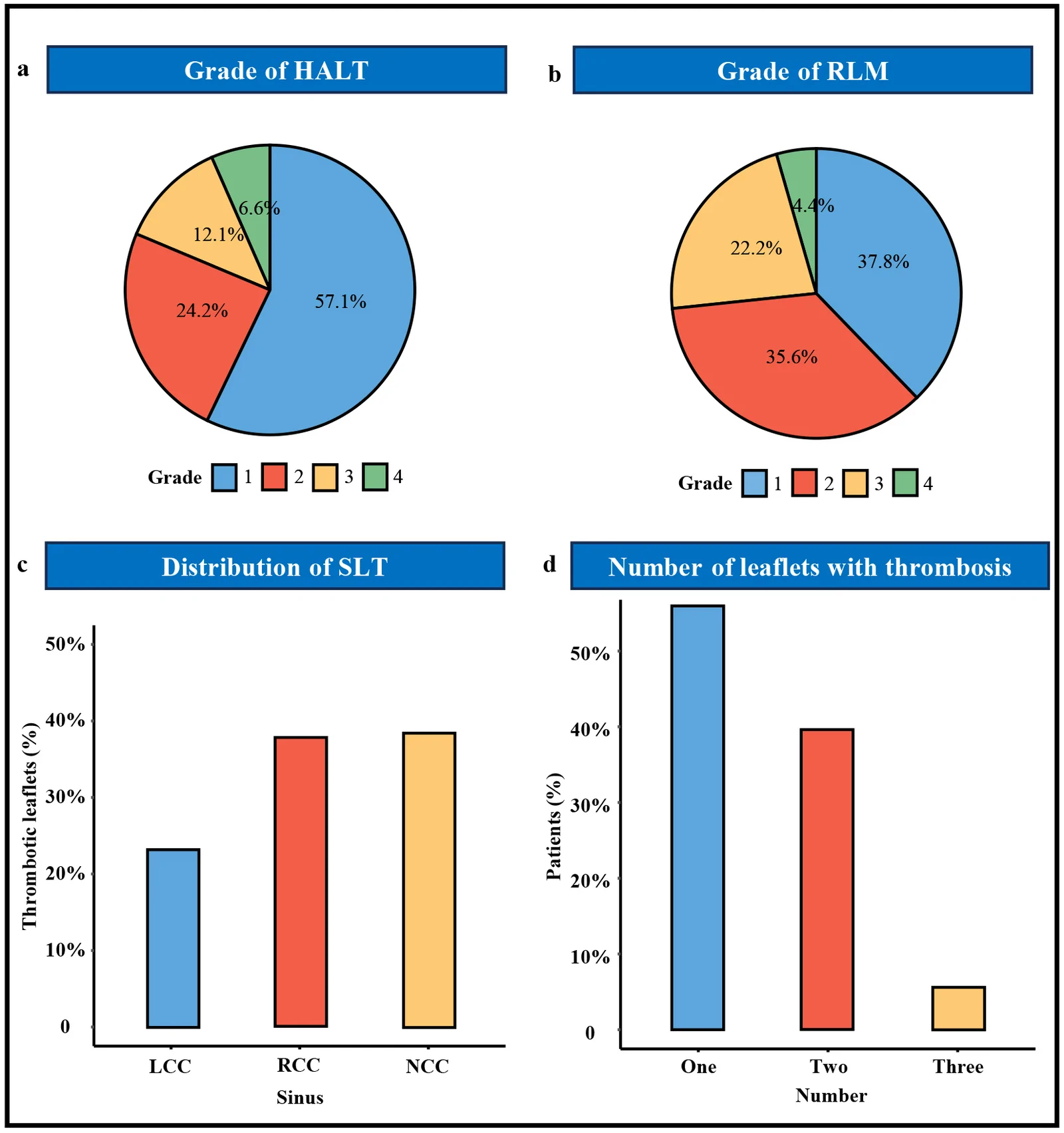
**Severity and distribution of subclinical leaflet thrombosis**. (a) Distribution of HALT grades. (b) Distribution of RLM grades. (c) Distribution of SLT in the aortic sinus. (d) The number of leaflets with thrombosis. Abbreviations: HALT, hypo-attenuated leaflet thickening; LCC, left coronary cusp; NCC, non-coronary cusp; RCC, right coronary cusp; RLM, reduced leaflet motion; SLT, subclinical leaflet thrombosis.

No statistically significant differences were observed in age, sex distribution, or incidence of bicuspid aortic valve between the SLT and no SLT groups (71.64 ± 8.82 years, 49.6% females, 50.5% bicuspid aortic valves vs. 72.95 ± 6.57 years, 42.9% females, 42.9% bicuspid aortic valves, respectively; all *p* > 0.05). The demographics of each group are detailed in Table 1.

Prosthetic valves with a skirt were used significantly more frequently in the SLT group than in the no SLT group (78.0% vs. 47.7%; *p* < 0.001) (Table 2). Patients in the SLT group had significantly larger prosthetic valve diameter (25.56 ± 2.65 mm vs. 24.22 ± 3.06 mm, respectively; *p* < 0.001), aortic valve annulus area (469.96 ± 100.77 mm^2^ vs. 426.90 ± 117.00 mm^2^; *p* = 0.003) and aortic valve waist area (448.08 ± 108.61 mm^2^ vs. 400.19 ± 130.40 mm^2^; *p* = 0.005) than those in the no SLT group. No significant differences in implant depth, taper of the transcatheter heart valve, and eccentricity (annulus, inflow, and outflow) were observed between the two groups.

**Table 2.** **Prosthetic valve characteristics and post-TAVR CT measurements**.

	Total cohort (N = 311)	No SLT (N = 220)	SLT (N = 91)	*p*-value
CT time (months)	1.07 [0.95, 5.85]	1.06 [0.96, 5.83]	1.07 [0.93, 5.88]	0.936
Prosthetic valve with skirt (n, %)	176 (56.59)	105 (47.73)	71 (78.02)	<0.001
Prosthetic valve diameter (mm)	24.61 (3.01)	24.22 (3.06)	25.56 (2.65)	<0.001
Taper of the THV (%)	82.76 (2.74)	82.89 (2.72)	82.43 (2.79)	0.259
Implant depth (mm)	6.65 (3.48)	6.64 (3.54)	6.66 (3.32)	0.974
Aortic valve inflow tract area (mm^2^)	464.00 (124.42)	455.75 (127.41)	482.08 (116.66)	0.182
Aortic valve outflow tract area (mm^2^)	425.14 (124.65)	417.87 (124.25)	442.14 (124.78)	0.162
Aortic valve annulus area (mm^2^)	439.54 (114.01)	426.90 (117.00)	469.96 (100.77)	0.003
Aortic valve waist area (mm^2^)	413.52 (126.37)	400.19 (130.40)	448.08 (108.61)	0.005
Annulus eccentricity (%)	14.10 (7.75)	14.59 (8.26)	12.92 (6.29)	0.099
Inflow eccentricity (%)	12.38 (7.29)	12.67 (7.80)	11.73 (6.03)	0.419
Outflow eccentricity (%)	10.01 (6.95)	10.22 (7.31)	9.52 (6.05)	0.469

Abbreviations: CT, computed tomography; SLT, subclinical leaflet thrombosis; TAVR, transcatheter aortic valve replacement; THV, transcatheter heart valve.

### 3.2 Logistic Regression Analysis for SLT After TAVR

Univariate logistic regression analysis revealed that SLT was significantly associated with prosthetic valve with skirt [odds ratio, 3.888; 95% confidence interval (CI), 2.251–6.964; *p* < 0.001] and larger prosthetic valve diameter (odds ratio, 1.167; 95% CI, 1.073–1.273; *p* < 0.001), as detailed in Table 3. Additionally, larger aortic valve annulus area (odds ratio, 1.003; 95% CI, 1.001–1.006; *p* = 0.004) and aortic valve waist area (odds ratio, 1.003; 95% CI, 1.001–1.005; *p* = 0.007) were also found to be significant predictors of SLT. In the multivariate logistic regression analysis, the prosthetic valve with skirt remained significantly associated with SLT (adjusted odds ratio, 2.999; 95% CI, 1.250–7.397; *p* = 0.015). In addition, anticoagulant therapy is also significantly associated with SLT (adjusted odds ratio, 0.462; 95% CI, 0.222–0.914; *p* = 0.032). In the logistic regression analysis of the four subgroups, compared to the group of prosthetic valves without skirt and not combined with PVL, the prosthetic valve with skirt and not combined with PVL group (adjusted odds ratio, 4.848; 95% CI, 1.713–14.690; *p* = 0.004) showed a significantly increased risk of SLT. No significant difference was observed in the group with prosthetic valve without skirt and PVL (adjusted odds ratio, 1.636; 95% CI, 0.591–4.579; *p* = 0.341) and prosthetic valve with skirt combined with PVL group (adjusted odds ratio, 3.173; 95% CI, 0.982–10.676; *p* = 0.056) (Table 4 and **Supplementary Fig. 1**). No significant multicollinearity was observed among the variables included in the multivariable model, as all VIF values were below 5 (**Supplementary Table 2**).

**Table 3.** **Univariate and multivariable logistic regression for subclinical leaflet thrombosis**.

	Univariate		Multivariate	
	OR (95% CI)	*p*-value	aOR (95% CI)	*p*-value
Prosthetic valve with skirt	3.888 (2.251–6.964)	<0.001	2.999 (1.250–7.397)	0.015
Prosthetic valve diameter (mm)	1.167 (1.073–1.273)	<0.001	0.975 (0.815–1.165)	0.782
Aortic valve annulus area (mm^2^)	1.003 (1.001–1.006)	0.004	1.005 (0.999–1.010)	0.092
Aortic valve waist area (mm^2^)	1.003 (1.001–1.005)	0.007	0.999 (0.994–1.003)	0.659
Bicuspid aortic valve, n (%)	0.736 (0.448–1.202)	0.223	0.930 (0.503–1.727)	0.818
Anticoagulation therapy, n (%)	0.544 (0.286–0.985)	0.052	0.462 (0.222–0.914)	0.032
Implant depth (mm)	1.001 (0.925–1.082)	0.974	0.964 (0.863–1.073)	0.509

Abbreviations: aOR, adjusted odds ratio; CI, confidence interval; OR, odds ratio.

**Table 4.** **Association between prosthetic valve with skirt, paravalvular leakage and leaflet thrombosis**.

			Univariate		Multivariate	
	N	Events, n (%)	OR (95% CI)	*p*-value	aOR (95% CI)	*p*-value
Skirt- PVL-	69	9 (13.04%)	ref	-	ref	-
Skirt- PVL+	51	10 (19.61%)	1.626 (0.605–4.434)	0.333	1.636 (0.591–4.579)	0.341
Skirt+ PVL-	92	38 (41.30%)	4.691 (2.156–11.157)	<0.001	4.848 (1.713–14.690)	0.004
Skirt+ PVL+	58	20 (34.48%)	3.509 (1.484–8.861)	0.005	3.173 (0.982–10.676)	0.056

Skirt- PVL-, prosthetic valves without skirt and not combined with PVL group; Skirt- PVL+, prosthetic valves without skirt and combined with PVL group; Skirt+ PVL-, prosthetic valves with skirt and not combined with PVL group; Skirt+ PVL+, prosthetic valves with skirt and combined with PVL group. Patients with missing postprocedural 1–6-month paravalvular leakage data were excluded from the skirt/PVL subgroup analysis. The group sizes and event counts shown in the table are based on the 270 patients included in the univariable and multivariable analysis. Abbreviations: aOR, adjusted odds ratio; CI, confidence interval; OR, odds ratio; PVL, paravalvular leakage.

## 4. Discussion

To the best of our knowledge, this is the first study to investigate the incidence and risk factors for SLT in patients undergoing TAVR using SEVs. Our primary finding was that SLT occurred in over one quarter of patients using SEVs. Notably, the implantation of SEVs with a skirt was identified as an independent risk factor for SLT, regardless of the presence or absence of PVL.

Previous studies have identified several potential risk factors for the development of SLT following TAVR, including postoperative prosthesis deformation, oversized prostheses, misalignment [4,15,19,20]. These studies provide evidence-based insights into the optimization of TAVR valve designs. SEVs are the most widely used type of TAVR valve in China and offer advantages such as repositionability, retrievability, and a larger effective orifice area post-procedurally. However, significant variability exists among various SEVs, including differences in size, tapering, and the presence of a skirt, which could impact the risk of SLT following TAVR.

This research comprehensively investigated potential risk factors that could influence SLT, including patient clinical characteristics, preoperative aortic anatomical features, and prosthetic valve characteristics. Our results indicate the use of skirted SEVs significantly increases the risk of SLT by more than threefold, regardless of the presence or absence of PVL. The skirt is a layer of material attached to the lower part of the valve. It is designed to enhance the seal between the valve and surrounding cardiac structures, thereby reducing the risk of PVL. Previous studies have demonstrated that skirting has a significant effect on reducing PVL [21]. However, our study has found that the skirt, whilst reducing PVL, increases the risk of SLT. Whether this is related to prolonged blood washout time in the neo-sinuses following TAVR, thereby promoting blood stasis, requires further investigation. Consequently, balancing the risk of PVL and SLT should be a critical consideration for manufacturers when designing prosthetic valves with skirts. Future research should systematically evaluate the effect of various skirt parameters (e.g., height, thickness, and material properties) on PVL and SLT. This will be instrumental in the development of safer and more effective prosthetic valves, thereby mitigating the risk of complications and improving patient outcomes.

The results of this study have a number of implications in terms of clinical significance and further research. First, it demonstrates the high incidence of SLT among patients undergoing TAVR using SEVs. Given the associations between SLT and valve dysfunction/adverse clinical outcomes, the incorporation of post-TAVR 4D-CT scans into routine clinical practice is crucial for the timely detection of SLT. Second, in patients at high risk of SLT, such as those using prosthetic valves with skirts, more aggressive follow-up and the use of potent antithrombotic medications are necessary to reduce the likelihood of SLT. Finally, our findings provide evidence-based support for the optimization of transcatheter heart valve designs.

## 5. Limitations

This study has several limitations that merit consideration. First, its retrospective design may limit the generalizability of the findings. Despite adjustments for known confounders, there remains the possibility of unaccounted factors. Second, while this study identified the SLT-associated risk factors, it did not identify the pathophysiological or blood-flow-related mechanisms underlying SLT development. This aspect warrants further exploration in future studies. Finally, the follow-up period was not long enough to correlate SLT with clinical events, such as stroke, long-term mortality, and rehospitalization for heart failure. Patient follow-up is ongoing, and long-term data may provide more definitive insights.

## 6. Conclusions

This study revealed that over one-quarter of patients undergoing TAVR developed SLT within six months following the SEV implantation. Using skirted prosthetic valves was independently associated with SLT in patients undergoing TAVR using SEVs. More extensive and prospective studies are necessary to validate these findings.

## Data Availability

The data will be available based on reasonable requests to the corresponding author.

## Abbreviations

CI, confidence interval; CT, computed tomography; HALT, hypo-attenuated leaflet thickening; OR, odds ratio; PVL, paravalvular leakage; RLM, reduced leaflet motion; SEV, self-expanding valves; SLT, subclinical leaflet thrombosis; TAVR, transcatheter aortic valve replacement.
