# Supplementary material for: Skirted Prosthetic Valves Are Associated With Increased Risk of Subclinical Leaflet Thrombosis Following Transcatheter Aortic Valve Replacement: A Multicenter Study
Source: Rev Cardiovasc Med. 2026 Sep 11;27(9):49694. doi: 10.31083/RCM49694 (PMC13653166; doi:10.31083/RCM49694)
Supplement: Supplementary file 1 [file 2153-8174-27-9-49694-s1.zip › Supplementary materials.docx]

**Supplementary Table 1: Valve Types and Classification by Outer Sealing Skirt.**

| **Valve type** | **N (%)** | **Outer Sealing Skirt** |
| --- | --- | --- |
| Vitaflow | 71 (22.83%) | Yes |
| Evolut Pro | 19 (6.11%) | Yes |
| TaurusElite | 86 (27.65%) | Yes |
| TaurusOne | 33 (10.61%) | No |
| Venus-A | 55 (17.68%) | No |
| Venus-A Plus | 47 (15.11%) | No |

**Supplementary Table 2: Testing for multicollinearity among variables.**

| **Variable name** | **VIF** |
| --- | --- |
| Prosthetic valve with skirt | 2.086 |
| Prosthetic valve diameter (mm) | 3.456 |
| Aortic valve annulus area (mm^2^) | 4.613 |
| Aortic valve waist area (mm^2^) | 3.614 |
| Bicuspid aortic valve, n (%) | 1.043 |
| Anticoagulation therapy, n (%) | 1.119 |
| Implant depth (mm) | 1.562 |

Abbreviations: VIF, Variance inflation factor.

**Supplementary Fig. 1: Forest plot for different paravalvular leak and prosthetic valves with skirt groups.**

Skirt- PVL-, prosthetic valves without skirt and not combined with PVL group; Skirt- PVL+, prosthetic valves without skirt and combined with PVL group; Skirt+ PVL-, prosthetic valves with skirt and not combined with PVL group; Skirt+ PVL+, prosthetic valves with skirt and combined with PVL group.

Abbreviations: PVL, perivalvular leakage.
